# Deep Neural-Assisted Flexible MXene-Ag Composite Strain Sensor with Crack Dual Conductive Network for Human Motion Sensing

**DOI:** 10.3390/ma18153537

**Published:** 2025-07-28

**Authors:** Junheng Fu, Zichen Xia, Haili Zhong, Xiangmou Ding, Yijie Lai, Sisi Li, Mengjie Zhang, Minxia Wang, Yuhao Zhang, Gangjin Huang, Fei Zhan, Shuting Liang, Yun Zeng, Lei Wang, Yang Zhao

**Affiliations:** 1 College of Water Conservancy and Hydropower Engineering, Sichuan Agricultural University, Ya’an 625014, China; fujunheng@sicau.edu.cn (J.F.); xiazichen@stu.sicau.edu.cn (Z.X.); 17323109510@163.com (H.Z.); dingxiangmou@foxmail.com (X.D.); laiyijie0322@163.com (Y.L.); l1952391446@163.com (S.L.);; 2 College of Mechanical and Electrical Engineering, Sichuan Agricultural University, Ya’an 625014, China; zhangmengjie@stu.sicau.edu.cn (M.Z.); 17711533620@163.com (M.W.); 18208316586@163.com (Y.Z.); 3 Aviation Engineering Institute, Civil Aviation Flight University of China, Chengdu 641450, China; huanggj@cafuc.edu.cn; 4 Beijing Key Laboratory of Lignocellulosic Chemistry, Beijing Forestry University, Beijing 100083, China; feizhan0605@bjfu.edu.cn; 5 College of Chemical and Environmental Engineering, Chongqing University of Arts and Sciences, Chongqing 402160, China; stliang@cqwu.edu.cn

**Keywords:** strain sensors, deep learning, heterogeneous surface strategy, MXene, silver nanoparticle

## Abstract

Developing stretchable strain sensors that combine both high sensitivity and a wide linear range is a critical requirement for health electronics, yet it remains challenging to meet the practical demands of daily health monitoring. This study proposes a novel heterogeneous surface strategy by in situ silver deposition on modified PDMS followed by MXene spray coating, constructing a multilevel microcrack strain sensor (MAP) using silver nanoparticles and MXene. This innovative multilevel heterogeneous microcrack structure forms a dual conductive network, which demonstrates excellent detection performance within GF_max_ = 487.3 and response time ≈65 ms across various deformation variables. And the seamless integration of the sensor arrays was designed and employed for the detection of human activities without sacrificing biocompatibility and comfort. Furthermore, by adopting advanced deep learning technology, these sensor arrays could identify different joint movements with an accuracy of up to 95%. These results provide a promising example for designing high-performance stretchable strain sensors and intelligent recognition systems.

## 1. Introduction

Motion tracking and whole-body limb reconstruction technologies have gained extensive attention in diverse fields ranging from encompassing high-precision motion recognition, performance analysis, and virtual/augmented reality technologies to human–computer interaction [1,2,3,4,5,6]. Wearable electronics integrated with strain sensors for three-dimensional limb tracking and biomechanical reconstruction have been proposed. And these advanced systems leverage inherent material flexibility and conformability to establish seamless epidermal interfaces, enabling precise monitoring of complex anatomical trajectories through continuous strain mapping. Furthermore, detection devices boasting superior electromechanical properties efficiently and swiftly translate deformation-driven alterations into electrical signals. Nonetheless, this sophistication necessitates stringent requirements for the detection capabilities of flexible sensor devices [7,8].

The optimization strategies for enhancing the detection capabilities of these flexible and wearable sensors could be broadly classified into conductive-network improvement, geometric design, and material-structure modification. The conductive metallic particles and two-dimensional materials, likely graphene and carbon nanotubes, along with other emerging materials, have garnered widespread application in strain sensor devices owing to their exceptional conductivity, remarkable flexibility, and convenient processing [9,10,11,12]. Furthermore, the conductive and detecting properties of the sensors can be significantly enhanced through strategic substrate design (such as polyethylene glycol and PDMS) and reinforcement of the conductive network, which enhances the uniform dispersion of conductive materials within the substrate and bolsters its high conductive feature, thereby offering an advanced approach to addressing the challenge of resistance escalation under tensile strains [13,14,15,16]. The bionic structural designs [17,18], which encompass the utilization of tricyclic minimal surfaces [19], caterpillar-inspired vertical and scale-like wings [20], bionic anisotropic structures [21,22], as well as biomimetic multilayer architectures [23,24,25], are the prevalent approach to enhancing detection sensitivity. Such strategies facilitate high-sensitivity motion detection by intricately manipulating the flexible substrate, albeit posing challenges in terms of complex processing methodologies and stringent accuracy requirements. And in light of research advances in sensor structures, numerous studies in recent years have focused on composite systems of nanomaterials and polymer matrices to optimize performance. The ultrasensitive flexible strain sensor based on graphene nanoplatelets doped with polyethylene glycol diglycidyl ether can monitor breathing in the Internet of Things with excellent tensile and compressive properties [26]. And the flexible piezoresistive strain sensors reinforced with silver nanowires and graphene nanoplatelets in PDMS are applied for human motion detection [27,28]. Moreover, the highly stretchable sensor reinforced with graphene in PDMS realized the function of monitoring human motion via electromechanical and complex impedance properties [29,30]. All demonstrate the advantages of nanomaterial–polymer composite systems in enhancing sensor sensitivity, flexibility, and functionality, providing strong support for fields such as wearable healthcare and the Internet of Things.

2D MXene, owning the high surface area, superior electrical conductivity, and active metal hydroxide sites, has emerged as a prominent material for applications in electromagnetic shielding, electrocatalysis, and flexible electronics [31,32]. However, MXene-based electronic devices, which employ spin-coating and vacuum filtration layering techniques for lamination, pose a potential risk of irreversible structural damage, resulting in the failure of conductive pathways under tensile strain, thereby limiting the measurement performance [33,34]. Additionally, the oxidative properties of MXene would cause the chemical degradation and reduce its functional properties [35]. To address these challenges, the proposition of constructing hierarchically interconnected dual conductive networks emerges as a promising approach. Although dual network structures based on conductive polymers like PPy and PEDOT/PSS have potential application prospects, there is still considerable room for improving their intrinsic conductivity and increasing their economic feasibility [36,37,38]. To tackle the vulnerabilities of MXene, particularly its susceptibility to oxidation and conductivity decrement due to strain deformation, a promising and strategic approach via leveraging the inherent chemical reactivity of MXene has emerged to establish robust MXene-metal dual-conductive network interconnections [39,40]. Silver nanoparticles through in situ synthesis are often employed for heterogeneous integration of flexible sensor devices due to their interfacial infiltration bonding and mechanical interlocking mechanism [41,42,43].

Herein, we successfully designed and fabricated a flexible and dual-conductive network strain sensor composed of MXene-Ag composite on the polydimethylsiloxane (PDMS) film, harnessing the synergistic interplay between chemical in situ reduction and electrostatic adsorption based on the heterogeneous surface strategy. The incorporation of silver nanoparticles (Ag NPs) significantly elevates the detection performance with a gauge factor of 487.3, a linear electromechanical response, and a detection threshold. The physicochemical feature of superior hydrophobicity, self-cleaning capabilities, exceptional conductivity, and electromagnetic shielding abilities was performed and indicated the prepared sensors have superior environmental suitability. Furthermore, this flexible electronic device demonstrates a remarkable 95% accuracy in recognizing human motion via integration with advanced deep learning algorithms, thereby presenting novel avenues for limb recognition in living organisms and fostering innovative designs in wearable strain sensor technology.

## 2. Materials and Methods

### 2.1. Materials

Ti_3_AlC_2_ powders (MAX) were purchased from Tongrun Info Technology Co. Ltd. (Langfang, China). Lithium fluoride, glucose, silver nitrate, polydimethylsiloxane (PDMS), hydrochloric acid, silver paste, and ammonia solution were purchased from Chengdu Hoboyo Technology Co. Ltd. (Chengdu, China). And all samples were used directly without further treatment.

### 2.2. Preparation of MXene

The synthetic method of MXene flakes was reported in previous studies [44]. In detail, 1 g of parent phase material Ti_3_AlC_2_ was gradually added into the solution containing 2 g of LiF and 40 mL of HCI (9 M), followed by about 10 min of standing to wait for the bubbles generated by the violent reaction to disappear and the mixture to cool down. Then, the mixture was reacted under continuous magnetic stirring for 48 h at 40 °C and was washed with distilled water under centrifugation several times until the pH value reached about 6. Subsequently, the washed residue was added into 100 mL of distilled water, ultrasonicated for 2 h, and centrifuged at 3500 r/min for 30 min. The supernatant was collected as the final suspension of MXene flakes with the concentration of 3 mg mL^−1^.

### 2.3. Preparation of PDMS

The PDMS monomer and curing agent were thoroughly mixed at a 10:1 ratio and then cured at 60 °C to form the desired structure. Subsequently, the PDMS film was treated with plasma for 15 min to achieve hydrophilic surface modification.

### 2.4. Fabrication of MAP Composite

The 1D conductor silver particles were synthesized via a chemical solution strategy. In detail, the glucose (5%) solution and silver nitrate solution (2%) were mixed in the presence of PDMS. The ammonia with a mass concentration of 7% is slowly dripped into the aforementioned mixture drop by drop until the solution becomes transparent at 60 °C for 30 min. Finally, the MXene solution (0.5 mL) was sprayed onto the surface of the Ag@PDMS surface, which was then naturally air-dried for 12 h and encapsulated with PDMS film.

### 2.5. Characteristics and FEM Simulation

The samples were used for structural analysis by X-ray diffraction (TDM-20, Dandong Tongda Technology Co., Dandong, China). Environmental Scanning Electron Microscopy (ESEM) (Quanta FEG 250, FEI, Morristown, NJ, USA) was employed for morphological observations, encompassing both surface and cross-sectional morphology of the samples. Energy-dispersive spectroscopy (EDS) was used to ascertain the coating distribution within the sample. The chemical composition was characterized by XPS (Thermo Escalab 250XI, Waltham, MA, USA). The conductivity testing was measured by the digital multimeter (RIGOL 300, RIGOL, Tianjin, China). Contact angle meters (JC2000D1, ZHONGCHENG, Shanghai, China) were employed for the characterization of hydrophobicity. The infrared thermal images were taken by the infrared imager (FLIR T420, FLIR, Portland, OR, USA). The SE of the MAP was tested by the waveguide method. The sample was placed between the transmitting terminal and the receiving terminal, and the vector network analyzer (MS4642, Anritsu, Morgan Hill, CA, USA) was used to record the current SE in real-time. To further understand the working mechanism of the MAP strain sensor, the geometry component system and the structural analysis system of COMSOL 6.1 were adopted.

## 3. Results and Discussion

### 3.1. Morphological Structure and Sensing Properties of MAP Strain Sensor

**Figure 1:**
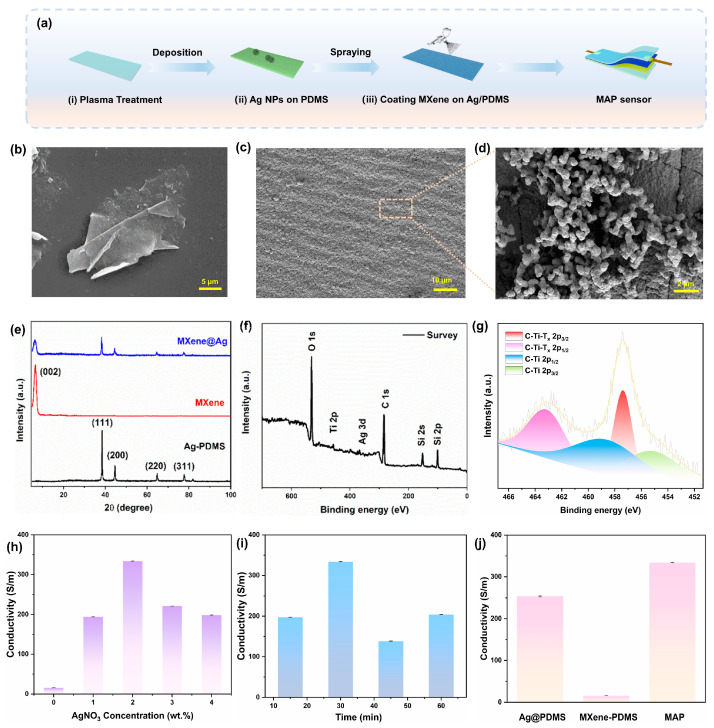
The structure and characteristics of the MAP composite. (**a**) Schematic diagram of the preparation and application of an MAP strain sensor. (**b**) SEM image of the MXene. (**c**,**d**) SEM images of the Ag layer. (**e**) XRD pattern of diverse samples. (**f**) XPS total survey spectrum of the MAP composite. (**g**) XPS pattern of the Ti 2p in MAP composite. (**h**,**i**) Concentration and reaction time on the conductivity of composite materials. (**j**) Conductivity of diverse sample materials.

The fabrication procedure of the MAP strain sensor comprising an MXene/Ag NPs dual-layered sensing layer and PDMS as the substrate is illustrated in Figure 1a. PDMS as the substrate layer was oxidized by plasma pretreatment for 15 min, resulting in the change from a hydrophobic to a hydrophilic surface, which could contribute to facilitating the in situ synthesis of silver nanostructures. Figure S1 illustrates the transformation in the optical photograph of the PDMS before and after pretreatment, demonstrating the successful plasma treatment. And the FTIR testing results in Figure S2 indicate that the Si-OH groups of the substrate changed significantly after pretreatment, which significantly influences the hydrophilic and hydrophobic characteristics of the substrate, favorable for subsequent in situ silver nanoparticle synthesis. The MXene/Ag NPs films were assembled on the prepared PDMS matrix. Thanks to the synergy between the strain resistance effect and the dual-layered conductive network architecture, the devised sensor boasts exceptional sensing capabilities, thermal management, and electromagnetic shielding, achieving multiple optimizations of sensitivity, stretchability, and multifunctional applications.

The MXene flakes solution was obtained from Ti_3_AlC_2_ powders via the LiF/HCl etching process [44,45]. Figure 1b and Figure S3 present scanning electron microscopy (SEM) images of the etched Ti_3_AlC_2_ at varying magnifications, which clearly demonstrate the transformation of Ti_3_AlC_2_ powders into a lamellar structure through solution etching. As illustrated by the transmission electron microscopy (TEM) analyses in Figure S4, the resultant structure exhibits a characteristic lamellar morphology with individual sheets measuring approximately 850 nm in size, and the Al layer in Ti_3_AlC_2_ is selectively etched [46]. Furthermore, Figure S5 displays the X-ray photoelectron spectroscopy (XPS) characterization of the synthesized MXene flakes for elucidating their chemical composition and valence states. Specifically, the Ti 2p spectrum reveals the distinct peaks corresponding to Ti-T_x_ at 452.95 eV, Ti-C 2p_1/2_ at 459.22 eV, and an additional Ti-C 2p_1/2_ peak at 453.90 eV. These Ti 2p peaks are indicative of the presence of the Ti_3_C_2_(OH)_2_ structure on the MXene surface, confirming the formation of hydroxyl functional groups. Notably, no spectral peak about aluminum is observed, affirming the successful etching process and subsequent preparation of MXene nanosheets.

Figure S6 illustrates the in situ synthesis of Ag NPs on the surface of substrates with various treatments, and the results indicate that the hydrophilic-treated substrate is more beneficial for the construction of the conductive network. The SEM micrograph of Ag NPs on the pretreated PDMS layer is shown in Figure 1c,d, and the silver particles fully encapsulate the pretreated PDMS surface, forming continuous nanoclusters that consist of aggregated Ag NPs visible on the surface of the silver layer. Figure S7a presents the elemental mapping results, revealing the corresponding elemental distributions. And the comprehensive elemental mapping spectra count for the Ag-PDMS composite is showcased in Figure S7b, which revealed a mass proportion of 93.28% for the Ag element, conclusively indicating the successful attachment of the Ag element to the treated PDMS surface. Compared to spray-coated layers prone to delamination due to weak interfacial adhesion, in situ-synthesized silver nanoparticles form densely packed architectures on flexible polymeric substrates, exhibiting superior interfacial adhesion strength capable of withstanding external stresses [47]. Figure S8 showcases the optical image of the MXene solution on the treated PDMS and Ag NPs modified PDMS matrix (Ag@PDMS). The MXene solution exhibits a contractile statement on the pretreated PDMS, preventing effective spreading. After the introduction of the Ag layer, the MXene is tightly stacked on the substrate, which is generated by the strong capillary force and strong hydrogen bond interaction between the MXene and the Ag nanoparticles during the solvent evaporation process [48,49,50,51]. Furthermore, Figure S9a displays the SEM image of MXene on the Ag@PDMS layer, which illustrates that the MXene layer is smooth and compact on the surface. And the cross-sectional image of the MAP composite with the PDMS encapsulation layer demonstrates a distinct structure with the layer-by-layer of PDMS-MXene/Ag-PDMS, indicating that the attachment of Ag does not compromise the structural integrity of the MXene layer. Notably, Figure S9b displays the thicknesses of the Ag conductive layer and the MXene electrode layer as approximately 1 μm and 10 μm, respectively.

The X-ray diffraction spectrum (XRD) testing results presented in Figure 1e elucidate the crystalline phase configuration of the fabricated samples, which align precisely with the distinctive peaks of face-centered cubic silver crystals (PDF#04-0783). These results affirm the successful immobilization of the synthesized silver nanoparticles onto the PDMS substrate. Furthermore, the exclusive presence of the characteristic peak of MXene, devoid of any other notable spectral features, underscores the comprehensive etching of the Al layer in Ti_3_AlC_2_, leading to the successful synthesis of the MXene monolayer, which is in agreement with the aforementioned SEM and TEM results. Regarding the composite material, the coexistence of characteristic peaks from both the silver layer and MXene signifies the successful integration of both components onto the PDMS surface. Furthermore, the chemical structure and valence states of the composite are elucidated by XPS analysis. The comprehensive survey of the XPS spectrum reveals the existence of C, O, Ti, Ag, and Si elements for the composite material (Figure 1f). The XPS spectrum pertaining to Ti exhibits signatures of C-Ti and Ti-Tx bonding, undeniably demonstrating the emergence of fresh functional groups on the MXene surface. The Ti XPS spectra derived from the MAP composite reveal distinct new peaks attributed to C-Ti-Tx bonding, firmly establishing the formation of C-Ti-Tx covalent linkages between MXene and the MAP composites (Figure 1g). And the distinctive peaks at 463.16 eV and 457.39 eV could be attributed to the 2p_1/2_ and 2p_3/2_ of C-Ti-Tx, respectively. The energy binding of 458.57 eV and 455.24 eV could belong to the C-Ti 2p_1/2_ and C-Ti 2p_3/2_, respectively. These peak intensities further indicated the presence of MXene on the composite surface. Compared with the high-resolution spectrum of Ti 2p in MXene, the Ti peak width of MAP is significantly increased, which may be attributed to the electronic perturbation of the carbon layer of MXene induced by the introduction of silver nanoparticles [52]. Additionally, Figure S10 demonstrates the XPS spectra of Ag with two pronounced peaks at binding energies of 373.08 eV and 367.87 eV, corresponding to the 3d_3/2_ and 3d_5/2_ orbitals of the silver element, respectively. Notably, the set of peaks centered at 373.90 eV and 366.24 eV should be attributed to Ag_2_O 3d_3/2_ and 3d_5/2_ orbitals [53]. This observation clearly indicates the presence of elemental silver form on the surface of the composite film. Figure S11 presented the thermogravimetric analysis (TGA) profiles of MXene/PDMS and MAP composites. At 800 °C, the MXene-PDMS composite exhibited a significant mass loss of 94%, whereas the MAP composite showed a lower mass loss of 79%, indirectly indicating that the silver nanoparticles occupy a mass fraction of about 15% in the composite materials.

Furthermore, the impact of solution conditions on the properties of the composite is examined. Notably, an increase in reaction concentration and duration leads to particle stacking, consequently diminishing the conductivity of the silver layer. As shown in Figure 1h,i, the insufficient reaction time of the silver-ammonia reaction and the low concentration of silver nitrate would result in the inadequate deposition of the silver layer, and its structure is discontinuous, obstructing the electron conduction path. However, with the prolonged reaction time or the high concentration of silver nitrate, the silver particles grow excessively and agglomerate, which would generate a porous structure, causing the increase in the electrical resistance. Therefore, the reaction concentration of 2 wt.% silver nitrate and a duration of 30 min are chosen for the subsequent preparation process. The impact of varying MXene contents on the electrical conductivity of the composites is explored in Figure S12, and the experimental outcomes indicate that the content of 5 mL MXene solution demonstrates superior adhesion and conductivity, underscoring its optimal concentration for the intended application. As shown in Figure 1j, the conductivity of different electrode layer materials is carried out, and the results showed that the sandwich structure composed of a silver layer and MXene has the best conductivity performance (334 S/m).

**Figure 2:**
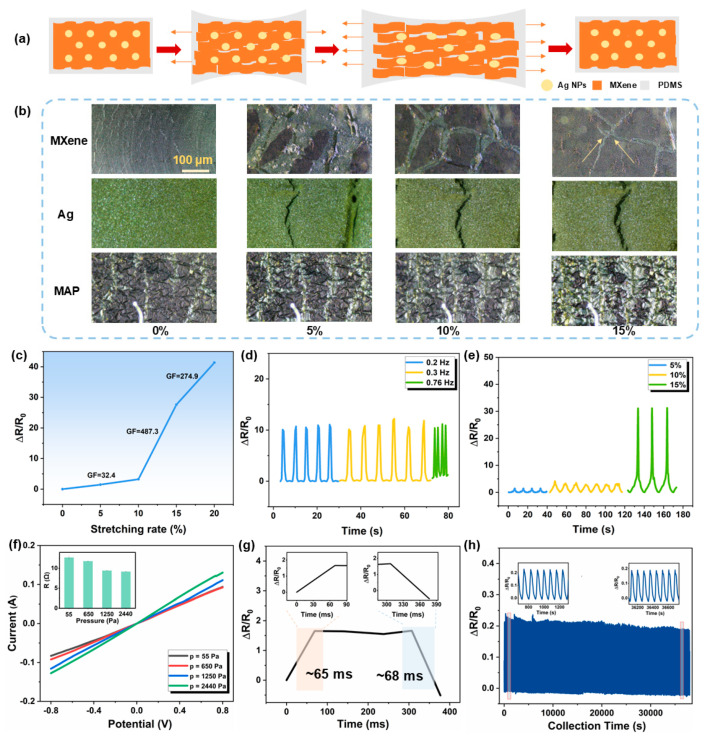
The electromechanical properties of the as-fabricated MAP sensor. (**a**) The deformation illustration of the strain sensor. (**b**) Optical photographs of crack morphology in MXene, Ag NPs, and MAP composite materials. (**c**) The sensitive testing of the MAP sensor. (**d**,**e**) The electrical resistance changes at different strain testing. (**f**) The curves under various pressures with applied voltage from −0.8 V to 0.8 V. (**g**) Response and recovery times of the sensor under 5% strain. (**h**) Testing of cycling stability of the MAP sensor.

The electromechanical properties of the flexible MAP strain sensor are critical to the overall sensing performance. Under applied strain, the alteration in the integrity of the conductive pathways resulted in the change in the electron transport path. Thus, when a strain sensor featuring diverse conductive pathways undergoes stretching, the strain imparted to the matrix is transferred to the brittle cracked layer via the interfacial phase. This applied force subsequently results in the change in the morphology and conductivity of the cracked layer, thereby affecting the overall conductivity of the strain sensor. Therefore, the structure and mechanical properties of the conductive network emerge as pivotal factors determining the morphology of the multi-conductive pathway sensors. As illustrated in Figure 2a, when the MAP sensor with dual conductive networks is stretched, the integrity of both the 1D conductive pathway composed of Ag particles and the 2D conductive networks with MXene film changes simultaneously, resulting in a synergistic effect that affects the overall sensor resistance. However, considering the differences in interfacial phase structure, the MXene conductive layer could become brittle and cracked upon experiencing external forces, modifying its electron transport properties even under minor deformation, which results in a reduction in the effective conductive pathway. The embedded Ag nanoparticles are employed as supplementary conductive networks, ensuring the continuity of the circuit.

Figure S13 and Supplemental Video S1 exhibit the dynamic working process of the MAP sensor under the applied stretching via the finite element modeling (FEM) strategy and the relative von Mises strain field of strain distribution as a function of displacement. To visualize the working process of the dual-conductance network composite sensor, three perspectives (side, top, and bottom) are used for the explanation. As shown in Figure S13i, with the stretching process, there is a large increment of strain distribution, especially in the edges of the geometry. Similarly, the strain distribution of the top surface in Figure S13ii exhibits that as strain is induced, the force is redirected from the perimeter of the geometry towards its center. However, the disparity in the modulus of elasticity among the materials within the network structure leads to a variation in the lateral distribution. Conversely, in the FEM of the bottom surface, the mechanical response is insignificant due to the remarkable ability of the PDMS substrate to endure substantial strains without significant deformation (Figure S13iii). In order to verify the electromechanical mechanism of the MAP composite, the morphology of cracks in various Ag NPs, MXene, and MAP composites was observed under optical microscopy as the strain increased. Figure 2b depicts that the microcracks that exist perpendicular to the stress direction in the film gradually separate when the sensor undergoes stretch. As tensile strain increased, the inter-segment spacing within all three samples exhibited progressive expansion. Notably, MXene films demonstrated significant crack propagation under strain. At 5% strain, the MXene layer experienced pronounced deformation, a phenomenon directly correlated with its high elastic modulus. When strain reached 15%, the conductive network in MXene underwent structural reorganization, transitioning to a point-to-point connection pattern. And the crack extension shows an irrecoverable state, leading to the failure of the conductive network. Similarly subjected to 15% strain, the standalone silver nanoparticle layer formed discrete point contacts. For the MAP composite, while fish-scale cracking developed in the surface MXene layer at 15% strain, the underlying silver layer became exposed through synergistic interfacial interactions. This unique structural response maintained lamellar continuity without observable cracks, preserving conductive pathway integrity and demonstrating enhanced strain tolerance capabilities.

Furthermore, the resistance changes and gauge factor (GF) sensitivity, a crucial parameter for assessing the sensing performance of the device, are carried out under various stretching strains. Typically, the gauge factor is defined as GF = (Δ*R*/*R*_0_)/*ε*, where *R*_0_ presents the initial resistance, Δ*R* denotes the resistance change before and after the stretching strain, and *ε* signifies the applied strain. Figure 2c depicts the resistance change ratio of the MAP sensor, wherein the augmentation of GF could be ascribed to the intensification of tensile strain. This elevated strain induces the formation of microscopic or macroscopic cracks between MXene and Ag NPs, thereby generating discontinuous conductive contact points, so that leads to a reduction in the internal conductive pathways, ultimately resulting in a decline in the overall conductivity of the composite. To assess the response cyclability of the sensor, it was subjected to varying strain frequencies (0.2, 0.3, and 0.76 Hz) while maintaining a consistent strain rate. The results, as presented in Figure 2d, reveal that despite the differences in strain loading frequencies, the relative resistance change in the prepared sensor converges and remains stable, demonstrating its adaptability to both rapid and gradual strain variations in practical applications. Figure 2e demonstrates the output signals at distinct strain rates (5%, 10%, and 15%), and the experimental results indicated the accuracy and repeatability in signal transmission under various tensile deformations of the MAP strain sensor.

The current–voltage characteristic curves of the MAP sensor under various pressures, as depicted in Figure 2f, exhibit a high degree of linearity, indicating the excellent ohmic contact properties. The inset reveals that as the pressure increases, the resistance of the sensor progressively decreases. This phenomenon can be attributed to the enhanced compaction of the layered MXene structure within the electrode layer against the Ag NPs in the interstices, resulting in closer integration and the formation of more conductive pathways. Figure 2g illustrates that the minimal response and recovery time of the sensor were 65 ms and 68 ms, respectively, and the equivalent response frequency is 14.7–15.4 Hz. Considering the frequency ranges of human motion, like skeletal muscle contractions, the MAP strain sensor was certainly favorable for real-time human motion and physiological monitoring as a wearable strain sensor [49]. Table S1 compares the detection performance of various strain sensors, from which it can be clearly seen that the MAP sensor in this study shows a relatively higher advantage in terms of sensitivity, while its response time is also slightly faster than that of the comparison objects, thus demonstrating overall excellent sensing and recognition performance. To further evaluate the durability and recyclability of the sensor for practical applications, the strain-sensing response during cycle testing was investigated. As described in Figure 2h, the cyclic results demonstrated that the MAP sensor consistently outputs signal waves with similar peak amplitudes and waveforms in each cycle, which indicated the resilience to continuous deformation and exceptional recyclability. And the variations in Δ*R*/*R*_0_ may be contributed to the modulus mismatching between diverse interfacial structures of the composite [54]. During continuous loading cycles, the sensitivity of the sensor decreases, and this behavior may be attributed to the disruption and rearrangement of the conductive pathways formed by the conductive phase within the elastomeric matrix. Under the action of strain, microcracks are generated inside the material and induce network rearrangement: the MXene and Ag at the cracks may be interrupted or form new contacts, leading to significant changes in resistance. During the fatigue process, both the continuous propagation of existing cracks and the constant generation of new cracks drive the continuous rearrangement of the conductive network—this causes the sensor sensitivity to fluctuate significantly in the initial stage due to the rapid formation of cracks; as the number of cycles increases, the elastic recovery and crack development gradually reach a dynamic balance, the network rearrangement tends to stabilize, and the variation range in sensitivity decreases accordingly. [55,56]. In addition, we have comprehensively investigated the influence of temperature and humidity on the sensor performance. As shown in Figure S14, the sensor was continuously monitored in an environment with 80% humidity, and its resistance change rate was tested every other day. The experimental results indicate that the prepared composite material exhibits good stability during the long-term testing process. In terms of temperature and sensing performance, since the constructed sensors are based on human motion detection, they are used in the temperature range near room temperature (≈10–40 °C), and subsequent electrical tests have shown that temperature variations have hardly any effect on the performance of the sensors. Also, the literature illustrates the negligible effect of temperature on the detection performance of wearable sensors [57].

### 3.2. Electromagnetic Interference Shielding and Self-Cleaning Properties of the MAP Strain Sensor

**Figure 3:**
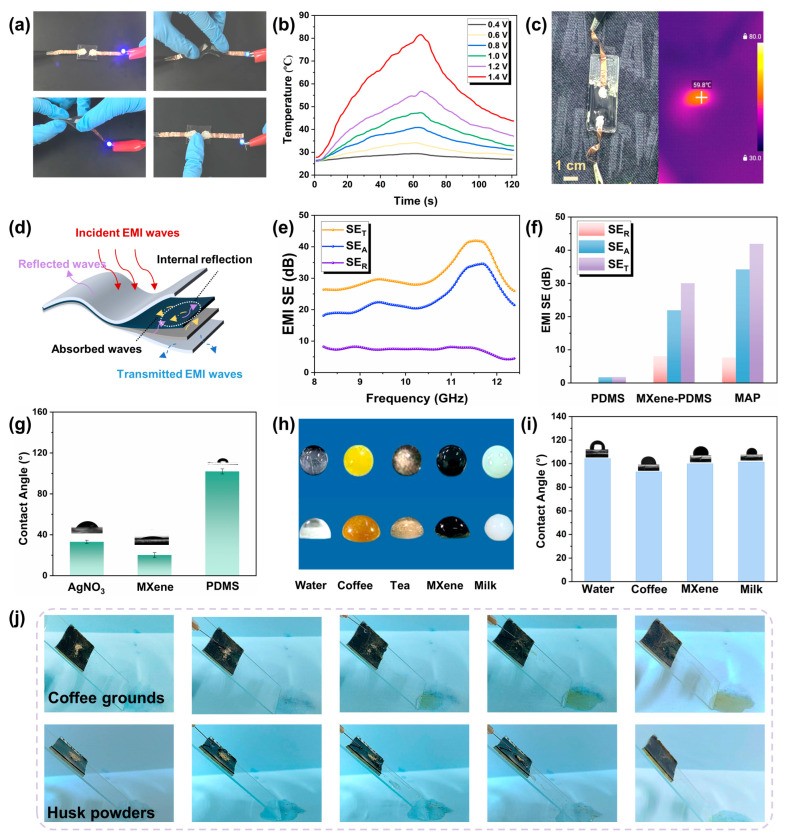
The characteristics of the MAP composite for the thermal/conductive, EMI, and hydrophobic properties. (**a**) The optical photographs of the MAP composite in the electrical circuit with various mechanical stimuli. (**b**) The electrical-thermal conversion with diverse applied voltage. (**c**) The infrared images of MAP composite on the clothes surface. (**d**) The electromagnetic shielding mechanism illustration for the MAP composite. (**e**) The electromagnetic shielding effectiveness property of MAP composite. (**f**) The SE_T_, SE_A_, and SE_R_ values of different samples. (**g**) The contact angle measurement of diverse pretreatment. (**h**) The optical photographs of colorful droplets on the MAP surface before and after pretreatment. (**i**) The contact angles of diverse droplets on the MAP surface. (**j**) The illustration of the self-cleaning property of the MAP surface.

To demonstrate the utility of the magnetic-conductive dual gradient structure, Figure 3 presents the results of the Joule effect, electromagnetic interference (EMI) shielding property, and self-cleaning performance of the composite films. The optical images of the MAP composite integrated into the circuit are presented in Figure 3a, and the experiments reveal that under various mechanical stimuli, including bending, twisting, or pressing, the circuit remained intact, thereby confirming the excellent electromechanical performance of the conductive pathways within the designed sensor. Herein, the Joule heating properties of the flexible strain sensor are investigated based on the superior conductivity. Figure 3b illustrates that the electric–thermal conversion performance of the MAP composite is evaluated with a fixed direct voltage from 0.4 V to 1.4 V, and the stable surface temperature was precisely captured by an infrared camera (shown in Figure S15). According to the specific data from the actual test results, as the DC voltage applied to the sensor gradually increases, the surface temperature of the sensor shows a significant upward trend, with the corresponding temperature changes being 24.9 °C, 34.2 °C, 40.9 °C, 47.4 °C, 56.8 °C, and 81.6 °C in sequence. This phenomenon clearly reflects the positive correlation between voltage regulation and the surface temperature of the sensor. Notably, at an external potential of 1.4 V, the surface temperature of the sensor attains 81.6 °C, exemplifying the remarkable electrical heating capabilities. Thus, Figure 3c exhibits that this flexible electronic sensor device could be mounted on the human body as an on-cloth Joule heater, which ensures stable thermal generation.

As the proliferation of various electronic devices, encompassing 5G micro-computers and smartphones, accelerates, the problem of electromagnetic wave pollution has grown increasingly prevalent, adversely impacting human health and the surrounding environment [58,59]. Therefore, considering their working scenarios, wearable electronics necessitate urgent incorporation of EMI shielding properties. The prerequisite for designing effective EMI shielding materials lies in their high electrical conductivity, as the conductive pathway for electrons is pivotal to the dissemination of electromagnetic waves. The dual conduction capability of the MAP composite indeed would make it the potential candidate to significantly enhance the EMI Shielding Effectiveness (SE), making it a highly promising material for applications requiring robust electromagnetic interference protection. Figure S16 demonstrates that an LED is remotely lit by a Tesla coil unit via electromagnetic waves and turned off when the MAP composite is held close to the coil, highlighting the satisfactory EMI shielding performance of the composite. To further evaluate the electromagnetic shielding capability of MAP composite, the EMI shielding performance of the MAP film in the frequency range of 8.2–12.2 GHz was demonstrated. The total EMI shielding effectiveness (SE_T_) encompasses both the absorption shielding efficiency (SE_A_) and reflection (SE_R_), which can be verified through the subsequent formula.

(1) $$SE_{T}\left(SE\right)=SE_{R}+SE_{A}$$

(2) $$SE_{R}=-10\log\left(1-R\right)$$

(3) $$SE_{A}=-10log(T/(1-R))$$

Figure 3d demonstrates the EMI shielding mechanism of the MAP composite films. When the electromagnetic wave reaches the surface of the MAP composite, most of the electromagnetic wave undergoes direct reflection due to an impedance mismatch between the composite surface and surrounding environment, and the incident wave interacts with the high concentration of carriers, resulting in strong reflection loss. The remaining incident waves that evade immediate reflection engage with the intricate dual-conductive network, characterized by a high electron density fostered by Ag NPs. This engagement results in multiple reflections and scattering events, during which the incident wave’s energy is transformed into thermal energy via conduction losses and dipole polarization mechanisms, contributing to absorption loss. Ultimately, a portion of the remaining electromagnetic waves persists in traversing the MAP composite [33]. The EMI shielding performance of MAP composite, pure PDMS, and Ag NPs-PDMS is demonstrated in Figure 3e,f and Figure S17. The testing demonstrates that the pure PDMS substrate lacks the capability to shield electromagnetic waves because of the insulating organic material of PDMS. The EMI shielding proficiency of PDMS films modified with MXene undergoes marked improvement, attributed to the exceptional electrical conductivity and unique layered architecture of MXene [60]. Furthermore, in MAP composites, the synergistic fusion of Ag NPs and MXene-modified PDMS surfaces yields a resilient dual conductive network, thereby conferring remarkable EMI shielding potential to the MAP composite films.

The inherent hydrophobic performance of the flexible substrate PDMS endows the strain sensor with exceptional hydrophobic and self-cleaning properties. As depicted in Figure 3g, the PDMS surface of the sensor exhibits a water contact angle of 101.98° ± 2.4°. After in situ synthesis on plasma-treated PDMS, the surface adorned with silver microporous plates demonstrates a reduced water contact angle of 33.15° ± 1.53°, facilitating MXene adhesion, which further lowers the angle to 20.29° ± 2.39° on the MXene surface. Figure 3h,i and Supplemental Video S2 (Supporting Information) showcase the non-wetting hydrophobic characteristics of the sensor surface against various liquids. The various droplets, such as water, coffee, milk, and MXene solution, remain spherical upon contact, underscoring the resistance to wetting. Following plasma treatment of the PDMS surface, droplets adopt an ellipsoidal shape, signifying a temporary reduction in hydrophobicity, which subsequently recovers over time. The protective PDMS layer on the sensor surface, with its hydrophobic nature, effectively repels a wide range of common liquids, safeguarding the internal electrode layer from damage, thereby enhancing the practicality of the MAP strain sensor for real-time data transmission in diverse liquid environments. Self-cleaning capabilities of the MAP composite are also conducted, as demonstrated in Figure 3i and Supplemental Video S3 (Supporting Information). Husk powders and coffee grounds are sprinkled on the encapsulating layer; the pollutants could be smoothly rinsed and slid off from the surface when washed with flowing water.

### 3.3. Application of Motion Sensing and Gesture Recognition with Deep Learning-Assisted Model

**Figure 4:**
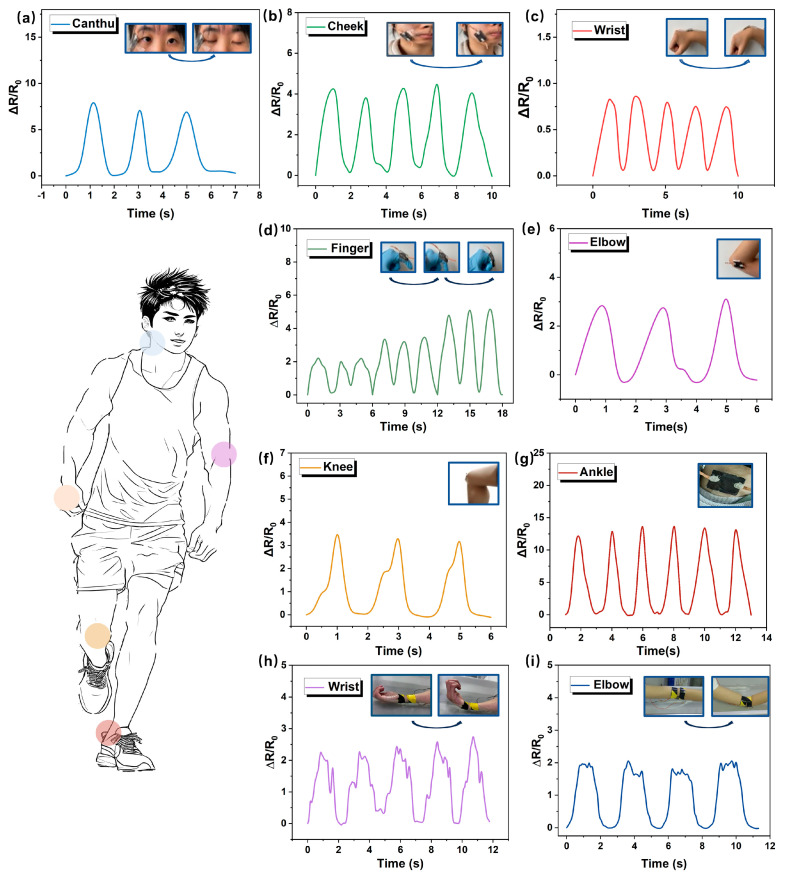
Application in real-time monitoring of human physiological signals. The different electrical resistance responses for (**a**) twinkling, (**b**) cheeking, (**c**) wrist bending, (**d**) finger bending, (**e**) elbow bending, (**f**) knee joint bending, and (**g**) ankle bending. The electrical resistance response underwater for (**h**) elbow bending and (**i**) wrist bending.

Due to the flexible, highly sensitive, and responsive characteristics, the MAP strain sensor exhibits excellent sensing performance and has great potential as a multifunctional sensor for human motion detection. To validate the practicality of the MAP strain sensor in human motion, the sensor was attached to diverse parts of human bodies for the full-range detection of human activities. Figure 4a demonstrates the capability of the MAP sensor to record the resistance variations around the eye corners induced by blinking. Upon systematically repeating the opening and closing of the eyes, the sensor registers a predictable pattern of increasing and decreasing resistance changes in the surrounding eye region, which is crucial for monitoring human sleep patterns. Figure 4b further illustrates the highly reproducible and stable resistance variation curve observed during cheek puffing movements, showcasing the robustness of the sensor. In monitoring more substantial movements, the sensor is mounted on the wrist to record the changes in current signals, and Figure 4c reveals the regularity in resistance changes when the wrist is positioned horizontally and then lowered. Additionally, the MAP sensor is adhered to the knuckle area to monitor the changes during the bending of a finger from 30° to 90° (Figure 4d and Supplemental Video S4). The sensing features of the MAP flexible sensor for larger deformations caused by movement of the elbow, knee, and ankle joints are recorded as the associated resistance changes and demonstrated in Figure 4e–g. In addition, the electrical signals of the elbow and wrist during underwater movement are monitored, demonstrating the excellent accuracy and hydrophobic performance of the sensors (Figure 4h,i). These experimental results indicate that the prepared sensor could sensitively detect and accurately record the change, demonstrating great potential as integrated wearable electronics for human health detection.

**Figure 5:**
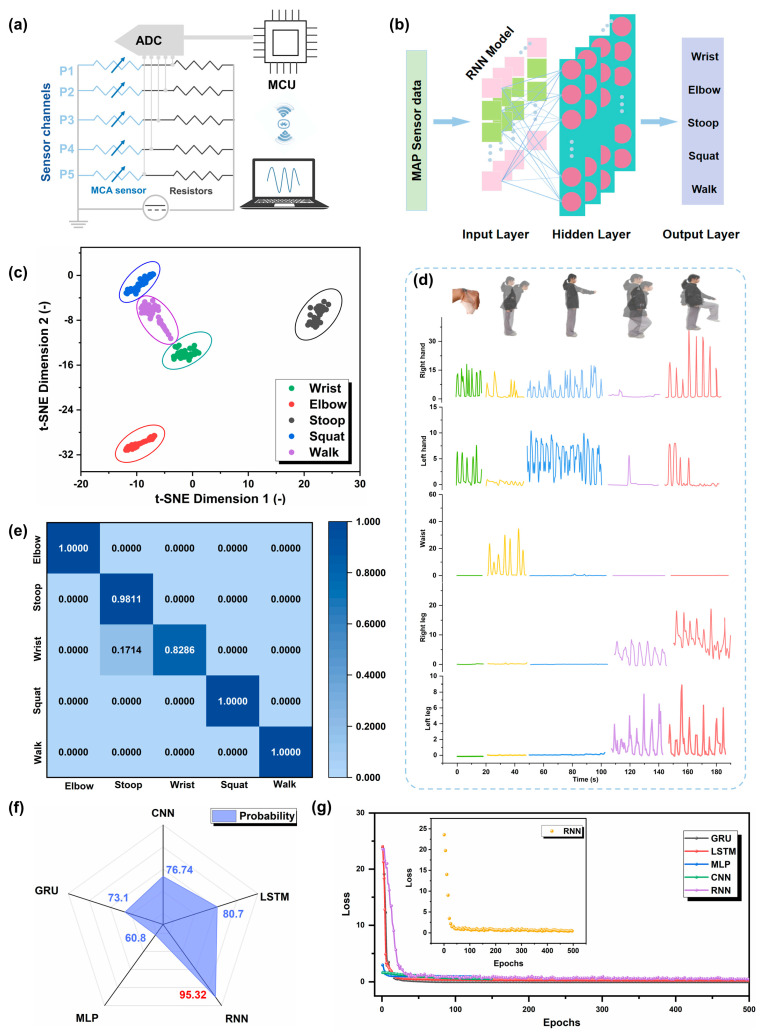
Deep learning-assisted MAP recognition system. (**a**) Schematic diagram of the equivalent circuit for the wireless sensor module. (**b**) Schematic illustration of the RNN deep learning algorithm, featuring a hybrid GRU-LSTM architecture with 1 GRU layer (128 units), 2 LSTM layers (64 and 32 units), and 3 Dropout layers (dropout rate 0.5). (**c**) t-SNE scatterplot of five body motions with two dimensionless parameters. (**d**) Signal outputs of five sensor arrays for body motion monitoring. (**e**) Identify the resultant confusion matrix, with the test set accounting for 20%. (**f**) Comparison of the performance of five diverse deep learning methods. (**g**) The loss curves of different methods in the 500 iterations training process, and the section is the loss curve of RNN.

This flexible strain sensor is capable of discerning the varied angular movements across different regions of the human body, offering insights into the broad applicability of flexible wearable sensors. However, in real scenarios, human movements are often intricate, rendering a single sensor type insufficient to identify complex and multi-joint movements. In order to improve the functional level of devices, the deep learning technique has recently been introduced into the sensor field [61]. In this study, the interactive system of the diverse array of MAP strain sensors networked with deep learning algorithms was constructed for recognizing complex human movement behavior (Figure 5a). With the help of the deep learning-assisted model, a large volume of real-world data could be analyzed simultaneously without the risk of missing or misinterpreting critical signals [62].

Bearing that in mind, the typical joints of human motions, including the wrist, elbow, waist, and limbs, have been chosen for gathering training data. A Recurrent Neural Network (RNN) is a specialized type of artificial neural network characterized by its internal circular connections, tailored explicitly for the processing of sequential data. Its defining trait lies in the presence of these circular connections, which facilitate the circulation of information within the network, enabling it to store and process sequential information efficiently. Figure 5b illustrates the schematic representation of the RNN algorithm. For the experiment, a Recurrent Neural Network (RNN) with six hidden layers is applied as the deep learning approach, where the dataset was partitioned into a training set and a testing set at a ratio of 80:20. The network utilizes Backpropagation Through Time (BPTT) for training, which is the RNN-specific variant of backpropagation. The input layer of the network comprises 20 neurons, each corresponding to the current resistance values, initial resistance values, resistance variations, and their rates of change for the limbs and waist. The neural network architecture incorporates three trainable hidden layers (1 GRU + 2 LSTM) with intermediate dropout layers, followed by an output layer with five nodes, each dedicated to recognizing five distinct actions, including wrist flexion, waist bending, elbow flexion, squatting, and walking. The network was trained using the Adam optimizer (initial lr = 0.005) with piecewise decay and He initialization, achieving a final training loss of 0.12. To ensure that the deep learning algorithm can effectively learn the specific signatures of human motions, we repeated each of the five actions, accumulating a comprehensive dataset of 8660 samples. Figure 5c showcases the t-distributed Stochastic Neighbor Embedding (t-SNE) scatterplot of multi-channeled sensor data in response to five body motions. Without the usage of images/video data, the RNN model was able to achieve an accurate identification for full-body motion classification. And the accuracy is defined in Equation (4), and the experimental results indicated that five clusters were formed after dimension reduction.

(4) $$Accuracy=100\%-\frac{1}{N}\sum_{i=1}^{i=N}\frac{\left|P_{i}-T_{i}\right|}{T_{i}}$$

where $P_{i}$ is the determined type of *i*th full-body motion, and $T_{i}$ is the recorded label of *i*th full-body motion in testing data [1]. After undergoing rigorous training and extensive testing, this system has showcased remarkable performance in both action recognition and prediction accuracy, exhibiting a high level of proficiency. As evidenced in Figure 5d,e, this system achieves exceptional differentiation among the five actions during the testing phase, with classification accuracies reaching 100%, 98.11%, 82.86%, 100%, and 100%, respectively. These experimental results emphatically highlight the vast potential of MAP strain sensors, particularly when integrated with deep learning algorithms, for sophisticated monitoring of human motion and facilitating seamless human–machine interaction. After training 500 iterations, the training loss reaches a desired state and the accuracy rate reaches 95.32%, and compared with other deep learning strategies, such as LSTM, GRU, CNN, and MLP, the recognition accuracy of the RNN method was superior, as showcased in Figure 5f. And Figure 5g exhibits the loss function curve in RNN, representing the trend of the training loss decreasing and saturating during the training process. Therefore, these results confirm that our motion recognition system, configured on the strain sensor array with the assistance of deep learning, has the potential to offer novel insights into the next generation of intelligent electronics.

## 4. Conclusions

In summary, we have designed and prepared a flexible, highly sensitive, and heterogeneous surface-structured MXene/Ag strain sensor for human movement recognition in this study. The incorporation of silver nanoparticles enhances the wettability of MXene on flexible PDMS substrates, addressing the challenge posed by the diminished electrical properties resulting from the oxidizability of MXene. And the dual conductive network of in situ synthesized silver nanoparticles and MXene assembly provides considerable electromechanical conductivity (334 S/m), detecting sensitivity (GF≈487.3), electromagnetic property, and hydrophobic performance. Moreover, this prepared sensor has achieved precise and swift detection of multiple motion patterns, as well as the accurate output signals of diverse joint activities and gestures under integrated sensor array and machine learning capabilities, achieving accuracies of up to 100%, 98.11%, 82.86%, 100%, and 100%, respectively. Finally, the proposed machine learning-assisted sensor network has demonstrated practical applications, opening a range of potential platforms for full-range and long-term health management, posture recognition, and human–machine interaction applications.

## Data Availability

The original contributions presented in this study are included in the article and Supplementary Materials. Further inquiries can be directed to the corresponding authors.

## Supplementary Materials

The following supporting information can be downloaded at: https://www.mdpi.com/article/10.3390/ma18153537/s1.
