# Supplementary material for: Deep Neural-Assisted Flexible MXene-Ag Composite Strain Sensor with Crack Dual Conductive Network for Human Motion Sensing
Source: Materials (Basel). 2025 Jul 28;18(15):3537. doi: 10.3390/ma18153537 (PMC12347900; doi:10.3390/ma18153537)
Supplement: Supplementary file 1 [file materials-18-03537-s001.zip › materials-3733812-Supporting Information.pdf]

SUPPORTING INFORMATION

Deep Neural-Assisted Flexible MXene-Ag Composite Strain Sensor with Crack Dual Conductive Network for Human Motion Sensing

Junheng Fu [1], Zichen Xia [1], Haili Zhong [1], Xiangmou Ding [1], Yijie Lai [1], Sisi Li [1], Mengjie Zhang [2], Minxia Wang [2], Yuhao Zhang [2], Gangjin Huang [3], Fei Zhan [4], Shuting Liang [5], Yun Zeng [1], Lei Wang [4,*] and Yang Zhao [1,*]

[1] College of Water Conservancy and Hydropower Engineering, Sichuan Agricultural University, Ya'an 625014, China; fujunheng@sicau.edu.cn (J.F.); xiazichen@stu.sicau.edu.cn (Z.X.); 17323109510@163.com (H.Z.); dingxiangmou@foxmail.com (X.D.); laiyijie0322@163.com (Y.L.); l19523914446@163.com (S.L.); yunzeng@sicau.edu.cn (Y.Z.)

[2] College of Mechanical and Electrical Engineering, Sichuan Agricultural University, Ya'an 625014, China; zhangmengjie@stu.sicau.edu.cn (M.Z.); 17711533620@163.com (M.W.); 18208316586@163.com (Y.Z.)

[3] Aviation Engineering Institute, Civil Aviation Flight University of China, Chengdu 641450, China; huanggj@cafuc.edu.cn

[4] Beijing Key Laboratory of Lignocellulosic Chemistry, Beijing Forestry University, Beijing 100083, China; feizhan0605@bjfu.edu.cn

[5] College of Chemical and Environmental Engineering, Chongqing University of Arts and Sciences, Chongqing 402160, China; stliang@cqwu.edu.cn

* Correspondence: leiwangns@bjfu.edu.cn (L.W.); yangzhao@sicau.edu.cn (Y.Z.)

CONTENTS

Supplementary Figures

Figure S1. the transformation in the optical photograph of the PDMS before and after plasma treatment.

Figure S2. The transformation of PDMS Fourier transforms infrared analysis before and after plasma treatment.

Figure S3. SEM images of the MXene.

Figure S4. TEM image of the MXene.

Figure S5. XPS images of the Mxene.

Figure S6. Optical photographs of in-situ growth of silver layer on PDMS surface before and after the plasma treatment.

Figure S7. EDS image and content of Ag@PDMS.

Figure S8. Optical photographs of MXene layer wetting on PDMS surface before and after the Ag layer modification.

Figure S9. SEM images of (a) surface and (b) section of MAP.

Figure S10. XPS pattern of the Ag 3d in MAP composite.

Figure S11. The TGA profiles of MXene/PDMS and MAP composite materials.

Figure S12. The influence of MXene concentration on the conductivity of composite materials.

Figure S13. The finite element analysis of proposed MAP strain sensor.

Figure S14. The resistance changes for long-time testing under humidity exposure (90%), and the results demonstrated excellent stability.

Figure S15. Infrared photographs of sensor surface temperature under fixed DC voltage of 0.4-1.4V.

Figure S16. Optical photo of LED remotely illuminated by Tesla coil.

Figure S17. EMI shielding performance of pure PDMS and Ag NPs-PDMS.

Supplementary Table

Table S1. The comparison of the sensing performance.

Supplementary Movies

Movie S1. The finite element analysis of proposed strain sensor.

Movie S2. The contact angle characteristics of diverse droplets on MAP surface.

Movie S3. The self-cleaning characteristics of MAP sensors.

Movie S4. The real time monitoring signal of MAP sensors integrated on the human joint.

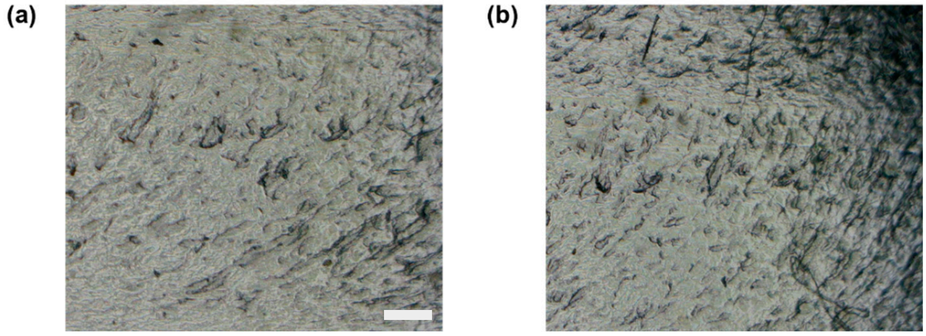

Figure S1. the transformation in the optical photographs of the PDMS before and after plasma treatment, and the scale bar is 50 μm.

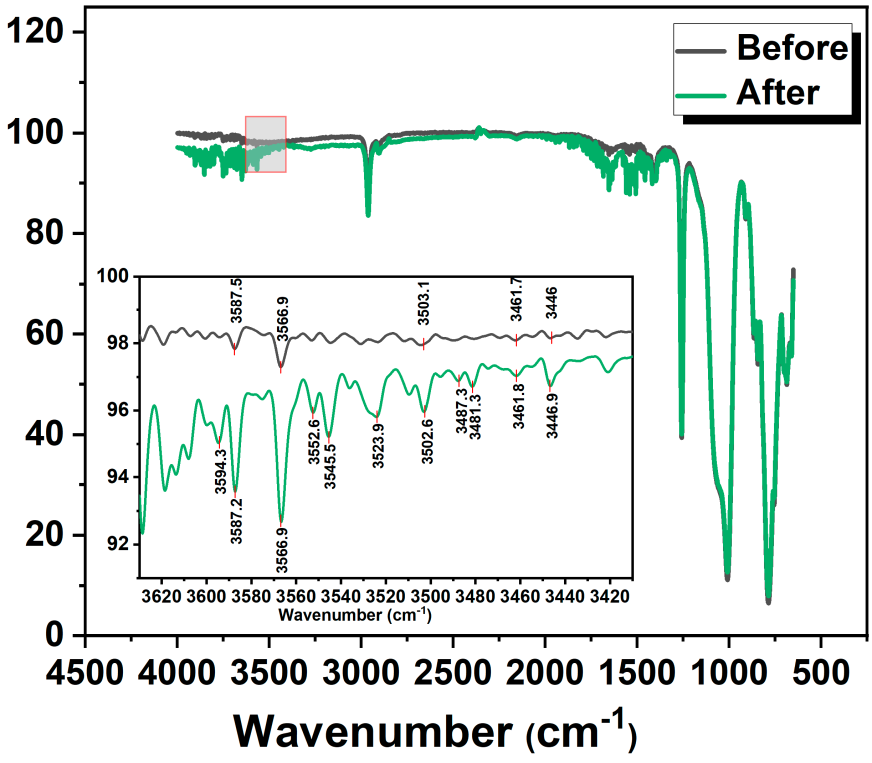

Figure S2. The transformation of PDMS Fourier transforms infrared analysis before and after pretreatment.

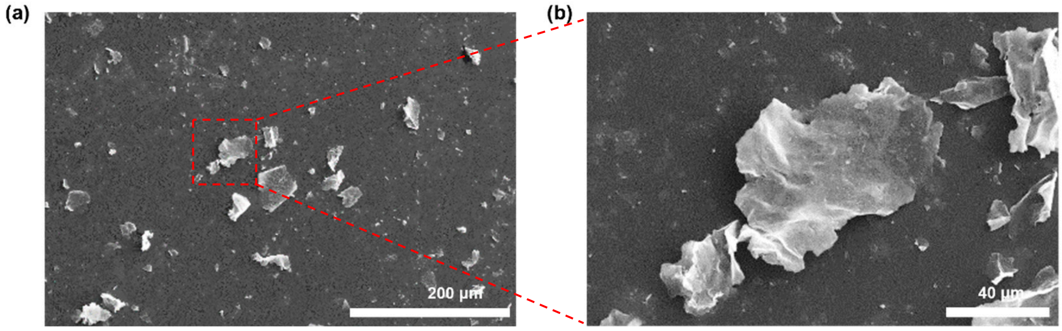

Figure S3. SEM images of the MXene, the sacle bar is 200 μm and 40 μm, respectively.

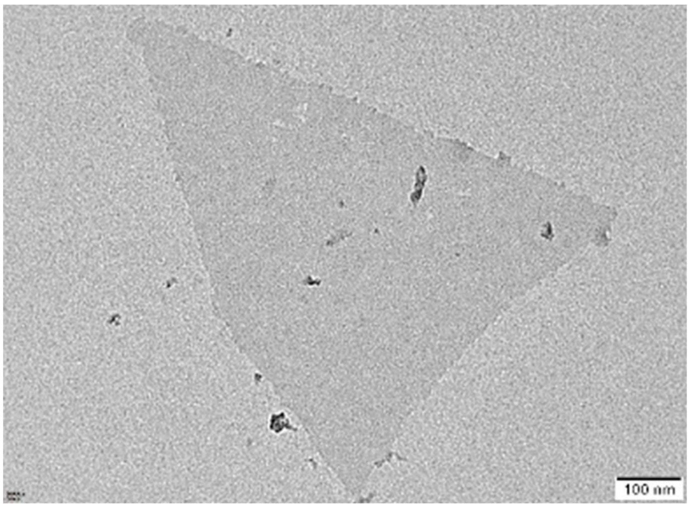

Figure S4. TEM image of the MXene.

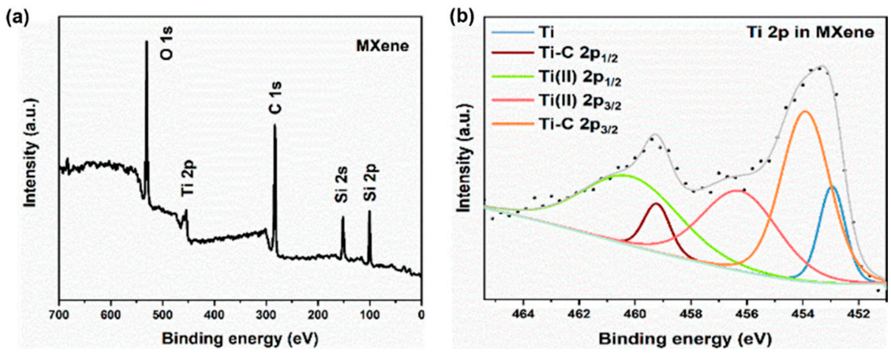

Figure S5. XPS of prepared MXene flakes. (a) XPS images of the Mxene. (b) Ti 2p spectrum.

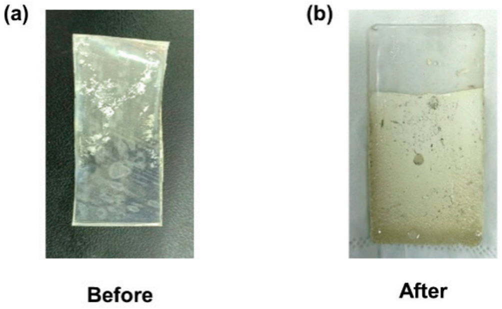

Figure S6. Optical photographs of in-situ growth of silver layer on PDMS surface before and after the pretreatment.

(a)

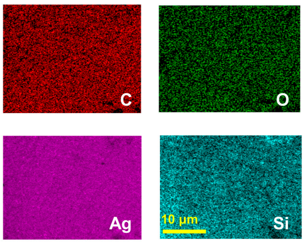

(b)

Total number of distribution spectra	
Element	wt.%
C	2.1
O	1.6
Si	3.02
Ag	93.28
Total	100

Figure S7. EDS mapping and content of Ag@PDMS sample.

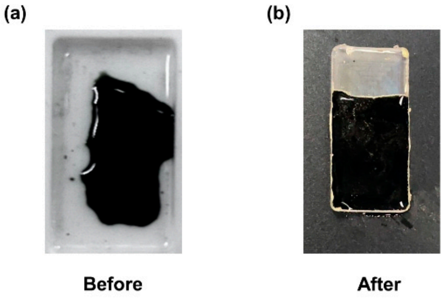

Figure S8. Optical photographs of MXene layer wetting on PDMS surface before and after the Ag layer modification.

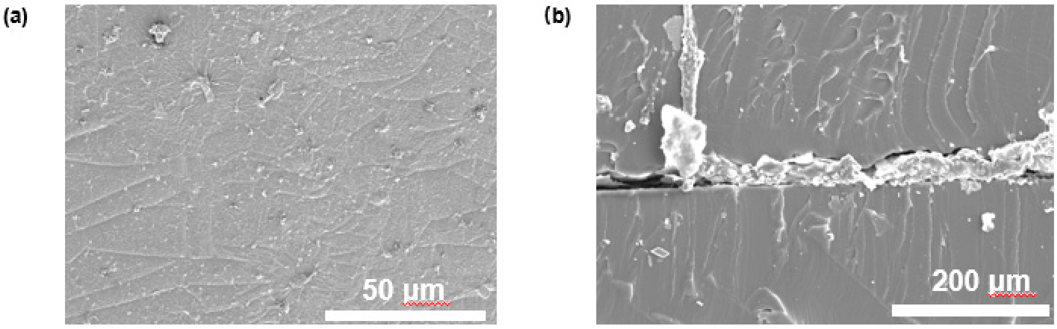

Figure S9. SEM images of (a) surface and (b) section of MAP.

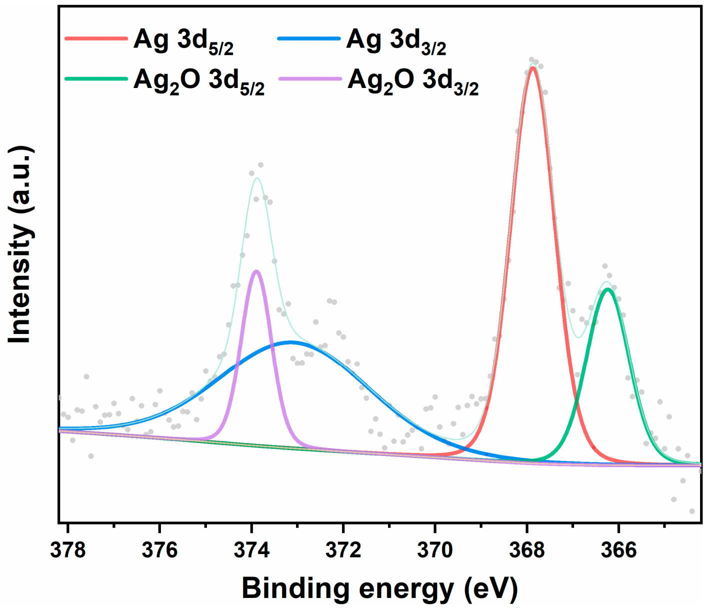

Figure S 10. XPS pattern of the Ag 3d in MAP composite.

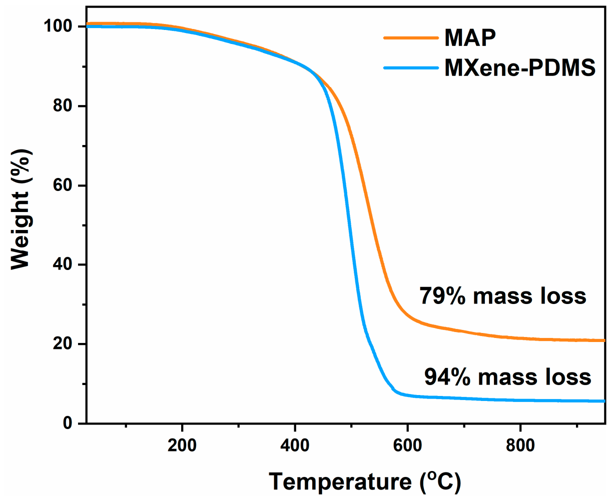

Figure S11. The TGA profiles of MXene/PDMS and MAP composite materials.

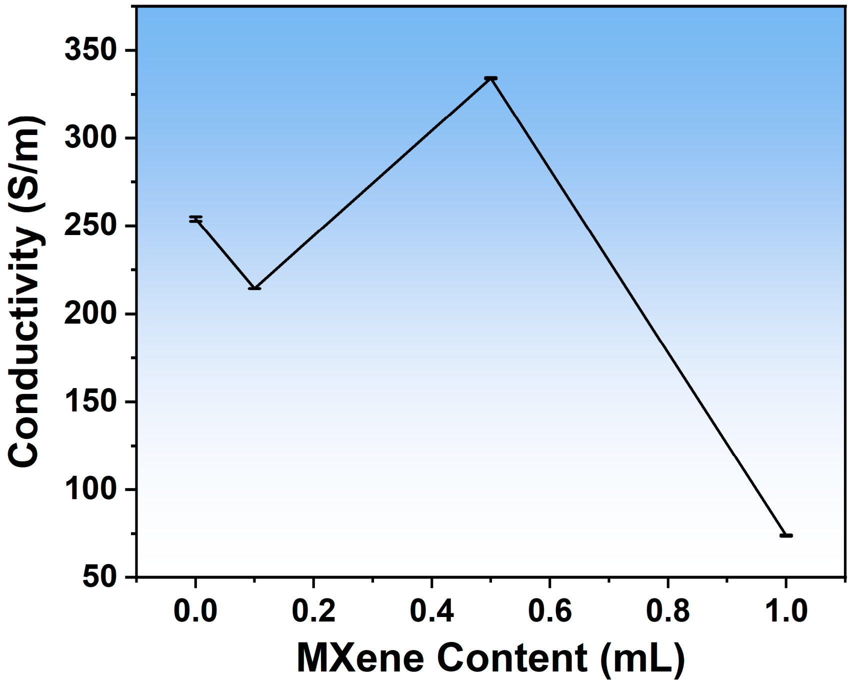

Figure S12. The influence of MXene concentration on the conductivity of composite materials.

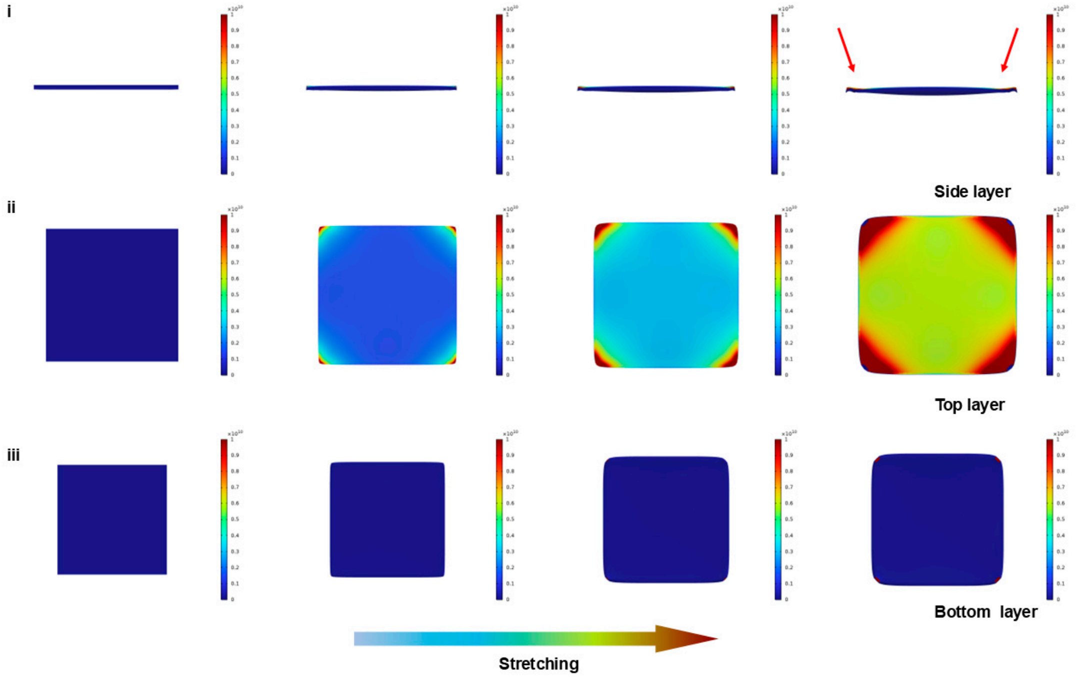

Figure S13. The finite element analysis of proposed MAP strain sensor (Unit: N m^{-2}).

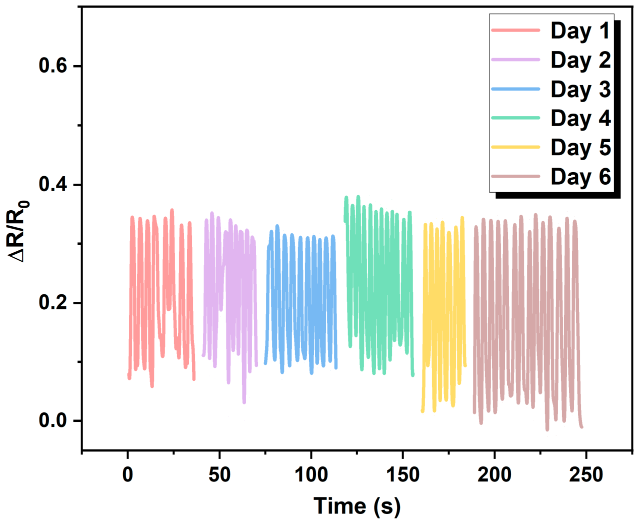

Figure S14. The resistance changes for long-time testing under humidity exposure (90%), and the results demonstrated excellent stability.

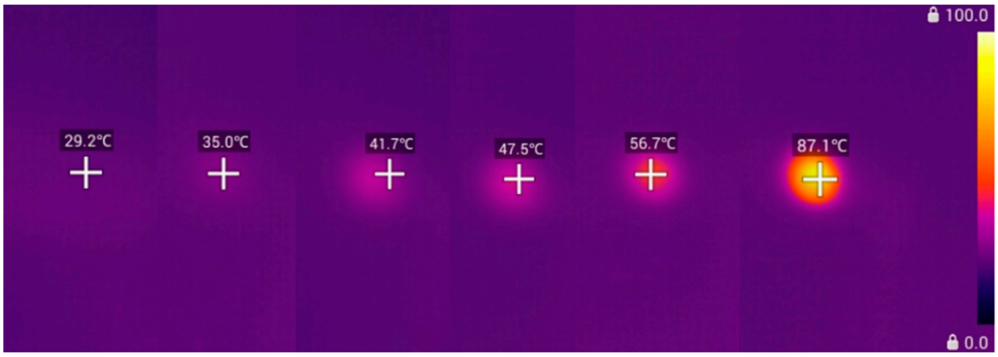

Figure S15. Infrared photographs of surface temperature of MAP sensor under fixed DC voltage of 0.4-1.4V after 60 seconds.

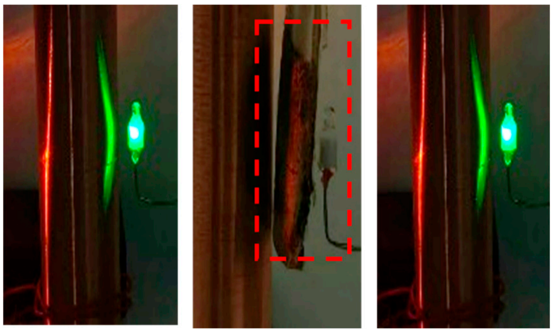

Figure S16. Optical photo of LED remotely illuminated by Tesla coil unit through electromagnetic waves.

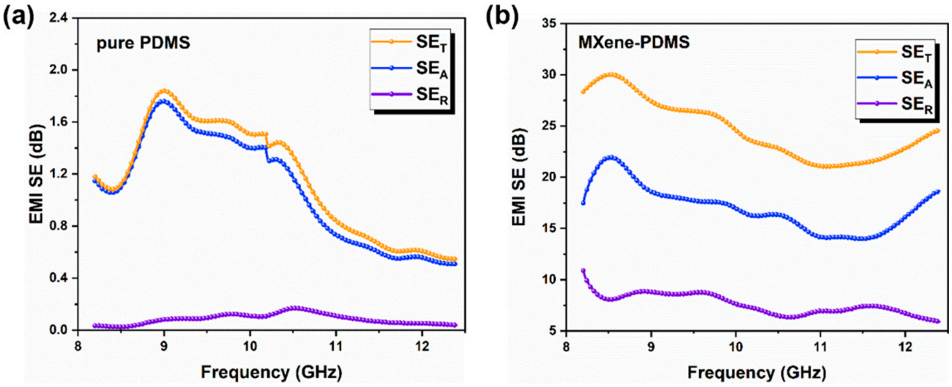

Figure S17. EMI shielding performance of pure PDMS and Ag NPs-PDMS.

Table S1. The comparison of the sensing performance.

Materials	Sensitivity	Response time	References
PCCN hydrogel	GF=0.864 (0<ε<100%)<br>GF=1.306 (100<ε<240%)	223 ms/232 ms	Y. Yu, Z. Zhou, H. Ruan, et al. Chem. Eng. J., 2025, 505, 158877.
SNF/PVA/MXene aerogel sensor	GF=3.43 (0<ε≤20%)<br>GF=7.86 (20<ε≤40%)<br>GF=24.61 (40<ε≤50%)	100 ms/200 ms	J. Chen, Z.-Z. Lu, J. Jin, et al. Surf. Interface, 2025, 62, 106220.
AgNW/MXene/PDMS composite films	GF=46 (0<ε≤34%)<br>GF=123 (35<ε≤50%)<br>GF=468 (51<ε≤68%)	200 ms	X. Bian, Z. Yang, T. Zhang, et al. ACS Appl. Mater. Interface, 2023, 15 (35), 41906-41915.
MXene/AgNWs/TPU strain sensor	GF=338.7 (0<ε<60%)<br>GF=5040 (60<ε<90%)<br>GF=33100 (90<ε<120%)	157 ms/203 ms	W. Qin, J. Geng, C. Lin, et al. J. Mater. Sci.: Mater. Electron, 2023, 34, 564.
MECCF sensors	GF=60 (0<ε<14%)<br>GF=210.6 (14<ε<30%)<br>GF=348 (30<ε<50%)	120 ms/200 ms	H. Lin, C. Zhang, N. Liao, et al. Compos. Pt. B-Eng., 2023, 254, 110574.
MXene/MWCNTs@TPU coaxial fibers	GF=4.6 (0<ε<25%)<br>GF=113.4 (25<ε<35%)<br>GF=553.9 (35<ε<45%)	200 ms/220 ms	L. Song, D. Kuang, J. Tang, et al. Sens. Actuators, A, 2025, 388, 116310.
MAP strain sensor	GF=32.4 (0<ε<10%)<br>GF=487.3 (10<ε<15%)<br>GF=274.9 (15<ε<20%)	65 ms/68 ms	This work
